# A Tribute to Dr. Chester Twitchell Stockwell

**Published:** 1915-11

**Authors:** James M’Manus


### A TRIBUTE TO DR. CHESTER TWITCHELL STOCKWELL.

BY DR. JAMES M'MANUS.

*At the Dedication of a Memorial in Forest Park, Springfield, Mass., October* 14, 1915.

Over forty years ago a bronze portrait statue of Dr. Horace Wells, the discoverer of anaesthesia, was placed in Bushnell Park, Hartford, the first public memorial to a professional man on record in the United States.

On the West Side high road to Springfield, six miles north of Hartford, on a plot of ground given by the Rev. William B. Carey, the traveler passes a memorial pedestal with a bronze tablet and a lantern, that every night brilliantly lights up the high road. The inscription can easily be read from sidewalk or carriage road. It tells of Dr. Horace H. Hayden, born in Windsor, Conn., a dentist, army surgeon, president and professor of the first dental college in the world and president of the first dental society, and is now referred to as "The Father of American Professional Dentistry."

We are here today to dedicate the third public memorial, all noticeably being located within twenty-five miles of Springfield, and each one dedicated to an ideal ethical professional dentist.

Historical, dental, medical and scientific works tell of Dr. Hayden and Dr. Wells, the work they accomplished and the blessings they bestowed. Many here present knew intimately Dr. Chester Twitchell Stockwell as a worthy, estimable citizen, a lovable man, companionable, sympathetic, helpful and thoroughly unselfish.

His early travel and residence in distant states, his education gained by mingling with and working with strangers at various occupations, his attendance at medical lectures, his journalistic life and work on the staff of a far distant western newspaper, all previous to his dental course of instruction, made him a welcome addition and member of the society of Springfield's ethical dentists forty-five years ago.

He was a skillful, sane and safe practicing dentist. He was a student and an investigator, a writer of ability and a scientist in the full meaning of that much-abused word. The Study Club of Springfield was a success, the *New England Dental Journal*, which he so ably edited, and the numerous practical and scientific papers he gave to the professions, through its columns, awakened keen interest and discussion in societies and scientific journals in this and foreign countries.

The numerous letters and telegrams received at the banquet tendered him at the Massasoit House, October 18, 1911, and the gathering of distinguished professional and business men present on that occasion, told how surely and how thoroughly he had gained the respect and affection of all who intimately knew him.

The one who planned this memorial surely was inspired, but only by the gracious permission and willing co-operation of the park board and the city officials of Springfield could it so successfully have been made "a thing of beauty and a joy forever." Springfield and Forest Park today open a new chapter in dental history, that will be read by professional and scientific men with interest throughout the civilized world.

Late in the summer of 1911, a pleasant afternoon tempted me to come to Springfield and ask Dr. Stockwell out for a slow ride through Forest Park, that I knew he was so deeply interested in and loved so fondly.

Memory, unbidden, often recalls interesting, inspiring and too often sadly pathetic incidents. But the future offers a new promise, and today we love Forest Park, for it assures us that as long as roses bloom, the young and aged alike, weary and thirsty, will drink from this beautiful memorial fountain, and Dr. Stockwell and his life work will not be wholly forgotten.

